# Enhancing MgO efficiency in CO_2_ capture: engineered MgO/Mg(OH)_2_ composites with Cl^−^, SO_4_^2−^, and PO_4_^3−^ additives†

**DOI:** 10.1039/d3ra04080a

**Published:** 2023-09-20

**Authors:** Hasanthi L. Senevirathna, Shunnian Wu, Cathie Lee, Jin-Young Kim, Sang Sub Kim, Kewu Bai, Ping Wu

**Affiliations:** a Entropic Interface Group, Engineering Product Development, Singapore University of Technology and Design 8 Somapah Road 487372 Singapore wuping@sutd.edu.sg; b Department of Materials Science and Engineering, Inha University Incheon 22212 Korea; c Institute of High Performance Computing, Agency for Science, Technology and Research Fusionopolis Way, #16-16 Connexis Singapore 138632 Singapore

## Abstract

The formation of a MgCO_3_ shell hampers CO_2_ capture efficiency in MgO. Our previous studies developed MgO/Mg(OH)_2_ composites to facilitate CO_2_ diffusion, improving capture efficiency. However, MgCO_3_ still formed along the interfaces. To tackle this issue, we engineered the MgO/Mg(OH)_2_ interfaces by incorporating Cl^−^, SO_4_^2−^, and PO_4_^3−^ additives. Novel MgO–H_2_O–MgX (X = Cl^−^, SO_4_^2−^, and PO_4_^3−^) composites were synthesized to explore the role of additives in preventing MgCO_3_ formation. MgO–Mg(OH)_2_–MgCl_2_ nano-composites displayed enhanced CO_2_ adsorption and stability. This breakthrough paves the way for effective bio-inspired strategies in overcoming CO_2_ transport barriers in MgO-based adsorbents.

## Abstract 2

Mg(OH)_2_–MgO interface for CO_2_ bypass.

## Graphical abstract

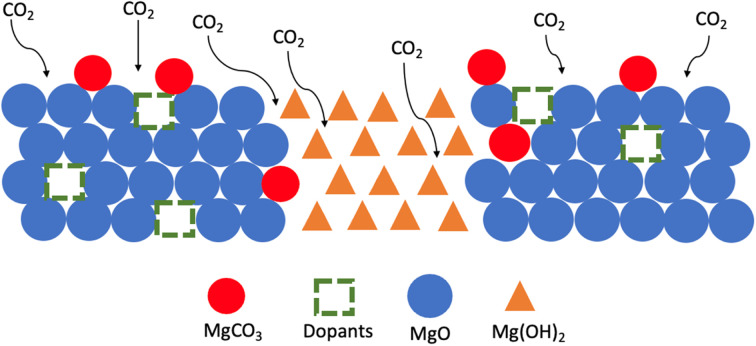

## Introduction

The escalating concentrations of greenhouse gases in the atmosphere have emerged as a pressing global concern, with far-reaching implications for all life forms on Earth. Among the greenhouse gases, the significant role of carbon dioxide (CO_2_) in causing global warming is widely recognized.^1–3^ Recent data indicates that atmospheric CO_2_ levels reached an average of 419.77 ppm by the end of February 2023, marking a substantial increase from 338.80 ppm in 1980.^4^ In response, scientists are actively seeking solutions to mitigate CO_2_ levels in the atmosphere. The synthesis of MgO powder from reject brine, which is a waste product from desalination plants, has been the subject of numerous experimental studies. Reject brine comprises large amounts of multiple anions and cations such as Cl^−^ (65593.1 ppm), Na^+^ (16124.3 ppm), Mg^2+^ (1679.0 ppm), SO_4_^2−^ (4322.6 ppm), Ca^2+^ (563.6 ppm) and many others.^5–8^ Typically, plants treat reject brine to remove these concentrated anions before releasing them back to the sea. However, there is a potential for extracting the ions along with MgO directly from reject brine. Consequently, it offers significant prospects for producing highly economic and environmentally friendly ion doped-MgO-based CO_2_ adsorbents on a large scale.^5,7,8^

Various materials are being currently involved for CO_2_ capture studies. They are mainly classified as polymeric membranes, ionic liquids (ILs), metal organic frameworks (MOFs), amine sorbents, and carbons. Even though they record high capture capacities, ability of disposing them to the environment without any harmful hazard is limited.^9^ Solid adsorbents have proven successful in trapping concentrated CO_2_ from industrial exhaust gases, offering an effective means of storage rather than direct emission into the environment.^10^ Several solid adsorbents with promising CO_2_ capture capacities have recently been proposed, with metal oxides emerging as particularly favorable candidates.^11–13^ Discovering a solid sorbent material capable of capturing CO_2_ at room temperature (RT) holds numerous advantages, including lower energy requirements, making the process economically viable for large-scale applications.^14^ Notably, magnesium (Mg)-based minerals present an abundant and environmentally benign option that can be produced on a significant scale at a relatively low cost.^15^ However, their inherent structural and morphological features restrict their adsorption capabilities, necessitating modifications to unlock their full potential.

MgO stands out to be a viable candidate for CO_2_ capture due to its higher theoretical CO_2_ capture capacity together with lower energy demand in regeneration in comparison to other metal oxides.^16^ Additionally, MgO is abundant on earth, of low cost and non-toxicity, and has a wide operating temperature (from room temperature to intermediate temperature).^17^ More, MgO exhibits significant CO_2_ chemisorption selectivity at temperatures below 200 °C.^10,16,18–21^ Despite its high theoretical CO_2_ capture capacity (1100 mg CO_2_/g sorbent), practical usage of MgO has been limited by a lack of active CO_2_ adsorption sites. Under dry, high-temperature conditions, MgO reacts with CO_2_, forming Magnesium carbonate (MgCO_3_).^10,15^ However, at lower temperatures and in moist conditions, MgO reacts with H_2_O to create intermediate products or hydrates that exhibit CO_2_ adsorption capabilities. Nevertheless, CO_2_ and H_2_O molecules may compete for adsorption sites on the MgO surface.^10^ Furthermore, the continuous exposure of the MgO surface to CO_2_ leads to saturation with MgCO_3_, impeding further CO_2_ insertion.^22^ Hu *et al.*,^10^ and Ruhaimi *et al.*,^23^ recently reviewed on the MgO based adsorbents for CO_2_ capture synthesized using various methods and at various conditions. The MgO synthesised by Zhao *et al.*,^24^ using combined surfactant assisted solvothermal or hydrothermal processes reported around 3.68 wt% of CO_2_ uptake below 350 °C. Elvira *et al.*,^25^ reported on MgO sorbent prepared using solution-combustion process and Ball milling method recording 1.61 wt% at 25 °C. Bhagiyalakshmi *et al.*,^26^ studied the MgO synthesized using template method at 25 °C recording about 8 wt%. A study by Song *et al.*,^27^ reported that the commercial MgO has the capture capacity of 0.88 wt%. They compared this with the porous structures calcined at different temperatures, where the best sample reported 3.6 wt% CO_2_ uptake. To overcome this challenge of low CO_2_ capture capacity of MgO itself, it is crucial to explore modified MgO-based materials and exploit their CO_2_ absorption mechanisms.

Recent studies have investigated the effects of Li, Na, and K nitrates on CO_2_ adsorption using commercial MgO powders at 300 °C. Optimization of the Li, Na, and K nitrate ratios for MgO doping enhances CO_2_ solubility in the salts and accelerates CO_2_ uptake, particularly when considering the influence of O_2_ concentrations on nitrites.^28^ Another approach involves the adoption of a solution combustion method by Elvira *et al.*, resulting in the production of MgO doped with urea at a 2 : 1 molar ratio of urea to magnesium nitrate. This strategy increases the CO_2_ adsorption capability from RT to 300 °C.^25^ Our research group has also reported significant progress in CO_2_ adsorption by developing bio-inspired MgO–Mg(OH)_2_ composites through a controlled steaming technique, albeit restricted to the MgO–H_2_O binary system. This technique has shown a remarkable improvement of approximately 25% in CO_2_ adsorption.^29^ Theoretical investigations on MgO–CaO composites incorporating Li, Na, K, and Rb promoters have revealed the successful utilization of Li dopants, which lead to changes in the properties of crystal surfaces, ultimately attracting CO_2_.^30^ However, the practical implementation of these active compounds raises concerns regarding environmental safety.

Over the years, various processes and methods have been employed to synthesize improved MgO-based adsorbents. These include sol–gel synthesis, hydrothermal synthesis, aerogel methods, ball-milling, template methods, and others, all contributing to the development of modified MgO materials for CO_2_ capture.^31–33^ Although these synthesis methods yield highly efficient MgO sorbents, they tend to be costly. In contrast, electrospinning offers a cost-effective approach to produce nanostructures and is renowned for its durability, adaptability, and scalability.^13^ Our research group has recently reported a study utilizing electrospinning to achieve mineralized CO_2_ capture from air at room temperature, using magnesium carbonate hydrate-based materials that achieved approximately 15.5 wt% of CO_2_ adsorption.^21^ This approach represents a smart strategy, bridging the gap between the current trial-and-error methods and a bioinspired rational design approach for developing MgO-based CO_2_ adsorbents.

In this research, our objective is to propose and validate two design rules using electrospinning synthesis techniques, aiming to develop a technique that incorporates both CO_2_-philic and CO_2_-phobic characteristics. (1) To design a highly efficient CO_2_ adsorbent, need to develop a tool that may couple the surface CO_2_-philic and CO_2_-phobic properties to balance the nucleation and transportation process during CO_2_ absorption. This is to eliminate the total obstruction of surface by the formed MgCO_3_ and allow new CO_2_ molecules to further absorbed through, as demonstrated in our previous study *via* a steaming process within the MgO–H_2_O binary system.^29^ The CO_2_-philic (MgO) and CO_2_-phobic (Mg(OH)_2_) domains in the sample further aid adsorbing additional fresh CO_2_ molecules, despite the persistence of intermittent MgCO_3_ precipitations neighboring MgO.^34^ CO_2_-philic parts are mainly for CO_2_ capturing while the CO_2_-phobic parts provide CO_2_ transport channels, which are unavailable in MgO. (2) To further mitigate the formation of MgCO_3_ along the MgO/Mg(OH)_2_ interfaces, our investigation focuses on ternary composites of Cl^−^, PO_4_^3−^, and SO_4_^2−^ doped MgO–H_2_O–MgX structures, where X represents 2Cl^−^, SO_4_^2−^, and 2/3PO_4_^3−^. Initially, the selection of anion X is based on observations highlighting their similar size and electronegativity, which result in comparable properties.^35^ Subsequently, we validate our selection through Density Functional Theory (DFT) calculations and chemical engineering modelling and simulation. This contribution demonstrates a novel strategy that employs magnesium-based dopants to achieve two key objectives: (1) maintaining a balanced CO_2_-philic and CO_2_-phobic function and (2) inhibiting the formation of MgCO_3_ along the composite interfaces in the design of ternary MgO–H_2_O–MgX systems. Our approach is guided by principles derived from quantum mechanics and thermodynamics and present improved capture capacity of 4.49 wt% for 10% Cl doped MgO by using electrospinning synthesis.

## Experimental

### Preparation of PVA/Mg(OH)_2_/MgCl_2_ solutions (1% MgCl_2_, 5% MgCl_2_, 10% MgCl_2_)

The solution with 1% Cl^−^ was prepared by dissolving 0.0025 g MgCl_2_ (Sigma-Aldrich) and 0.2475 g Mg(OH)_2_ (Sigma-Aldrich) in 5 mL acetic acid (Scharlau) *via* sonication in a water bath at 40 °C for 1 h. Then, the solution was mixed with the 5% w/w PVA (polyvinyl alcohol) as prepared above at a volume ratio of 15 : 100 (0.750 mL to 5 mL), with further sonication for 20 min at 40 °C. The 5% Cl^−^ solution was prepared by 0.0125 g MgCl_2_ and 0.2375 g Mg(OH)_2_. The 10% Cl^−^ solution was prepared by 0.025 g MgCl_2_ and 0.225 g Mg(OH)_2_ followed by the same procedure as stated for the 1% Cl^−^ solution.

### Preparation of PVA/Mg(OH)_2_/MgSO_4_ solutions (1% MgSO_4_, 5% MgSO_4_, 10% MgSO_4_)

The solution with 1% SO_4_^2−^ solution was prepared by measuring the similar weights mentioned in preparation of PVA/Mg(OH)_2_/MgCl_2_ solutions, but instead using MgSO_4_ (Macklin). Briefly, 0.0025 g MgSO_4_ and 0.2475 g Mg(OH)_2_ (Sigma-Aldrich). The measured samples were dissolved in 4 mL acetic acid (Scharlau) and 2 mL deionized water *via* sonication for 1 h at 40 °C. The solution was then mixed with 5% PVA 15 : 100 ratio as mentioned in preparation of PVA/Mg(OH)_2_/MgCl_2_ solutions. The 5% SO_4_^2−^ and 10% SO_4_^2−^ solutions were prepared by following a similar procedure stated for the 1% SO_4_^2−^ solution.

### Preparation of PVA/Mg(OH)_2_/Mg_3_(PO_4_)_2_ solutions (1% Mg_3_(PO_4_)_2_, 5% Mg_3_(PO_4_)_2_, 10% Mg_3_(PO_4_)_2_)

The solution with 1% PO_4_^3−^ solution was prepared by measuring the similar weights mentioned in preparation of PVA/Mg(OH)_2_/MgCl_2_ solutions, but instead using Mg_3_(PO_4_)_2_ (Acro-Organics). The measured weights were dissolved in 7 mL of 5 mol dm^−3^ acetic acid (Scharlau) *via* sonication for 1 h at 40 °C. Then the aqueous solution was added to 5% PVA with a ratio of 3 : 28 (0.750 mL to 7 mL), with further sonication in a water bath at 40 °C for 20 min. The 5% PO_4_^3−^ and 10% PO_4_^3−^ solutions were prepared by following a similar procedure stated for the 1% PO_4_^3−^ solution.

### Synthesis of Cl^−^, SO_4_^2−^ and PO_4_^3−^ doped MgO samples

The electrospinning process was carried out by following the parameters stated in our previous study.^21^ Carefully collected fiber layer dried at 60 °C for 48 h, and then calcined at 300 °C for 2 h in a box furnace (Anhui Haibei 1100 model).

### Characterization methods

The surface topography and morphology of nanomaterials were examined using field emission scanning electron microscope (FESEM), JEOL JSM-7600F FESEM. A small layer of gold was sputtered on to the sample surface before SEM analysis to encourage secondary electron emission, ensure uniform specimen conductivity, and offer a homogeneous surface for analysis. Thermogravimetric analysis (TGA) was performed in a CO_2_ atmosphere using Q50-TA Instrument to determine the CO_2_ adsorption capacities and long-term stability of the synthesised materials. X-ray diffraction (XRD) patterns of synthesized materials were obtained *via* Bruker D8 Advance XRD, using nickel-filtered Cu-Kα radiation (*λ* = 0.15418 nm) operated at 25 mA and 40 kV, with a step size of 0.01 (2*θ*) employing Cu Kα radiation (*λ* = 1.5406 Å). Gas sensing measurements were carried out using Keithley 2400 Source meter with a total flow of 500 SCCM. The features of gas sensing were studied in a horizontal quartz heating chamber. Data from dynamic sensing were captured with a constant DC bias of 1 V. Target gas CO_2_ (*R*_g_) and air (*R*_a_) output resistances were measured, and the sensor's response was calculated as *R*_a_/*R*_g_. Brunauer–Emmett–Teller (BET) analysis carried out using BET ASAP 2020 Specific Surface Analyzer to determine the surface area and pore size distribution of the samples. BET analysis also provide an analysis of adsorption–desperation isotherms for the samples.

## Results and discussion

### Structural properties

Structural characteristics of synthesized samples were observed using XRD analysis. The main peaks of the Cl^−^-doped samples matched with characteristic peaks of MgO, indicated by ‘#’ (ICDD 00-045-0946) and pure Mg(OH)_2_ (ICDD 00-044-1482).

**Fig. 1:**
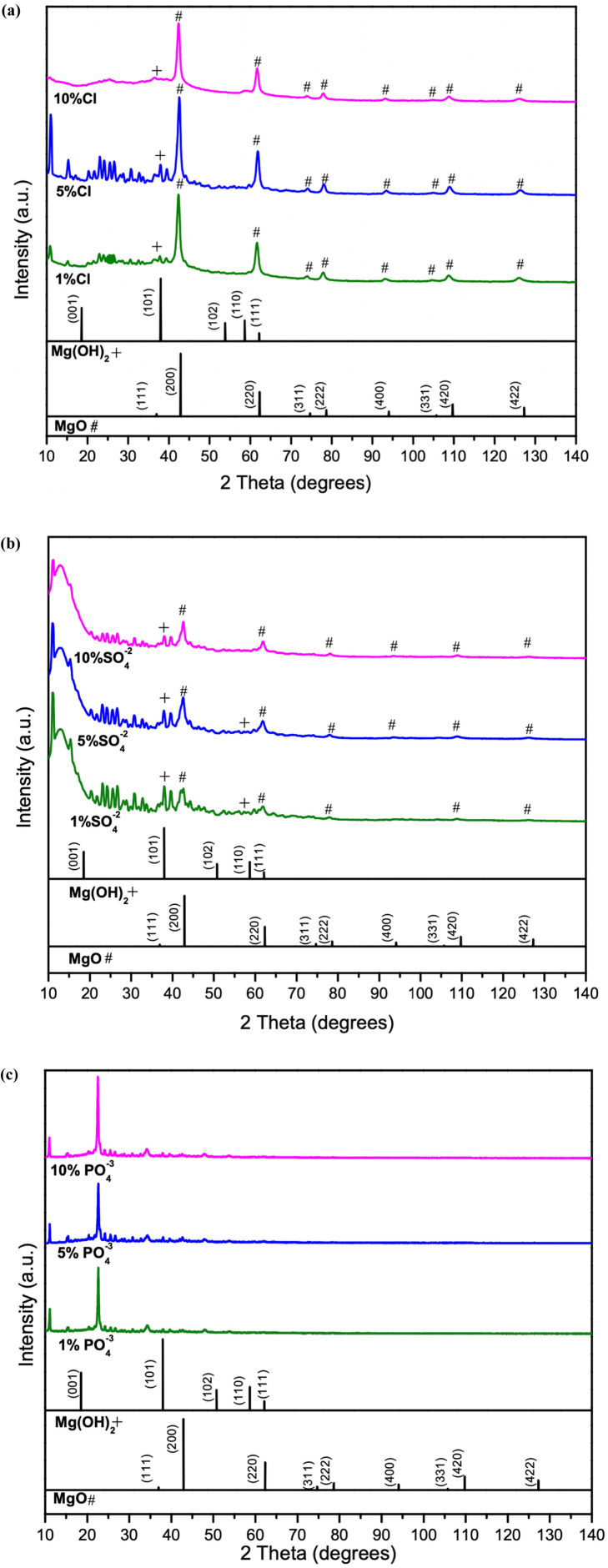
Comparison of XRD data of 1%, 5% and 10%, of (a) Cl^−^ doped (b) SO_4_^2−^ doped, and (c) PO_4_^3−^ doped samples.

MgO(111), MgO(200), MgO(220), MgO(331), MgO(222), MgO(400), MgO(420), MgO(422) and Mg(OH)_2_ indicated by ‘+’ (101) from 35° to 140° as shown in Fig. 1a. From 10° to 35°, peaks mainly belong to multiple hydrides shown in Fig. S1a,† it is evident that the peaks are belongs to multiple hydrides of chlorine, as magnesium chlorate hydrate (Mg(ClO_4_)_2_·*x*H_2_O) (ICDD 00-031-0789), magnesium chloride carbonate hydrate (Mg_2_Cl_2_CO_3_·7H_2_O) (ICDD 00-021-1254) and magnesium chloride diethylene glycol (C_8_H_20_Cl_2_MgO_6_) (ICDD 00-031-1763). The sharp diffraction peaks in Fig. 1a, 1% Cl^−^ doped sample indicate the better crystallinity. It is evident that increase in dopant percentage, results poor crystallinity in samples. In addition, the distinctive MgO peaks shifted toward the lower angles depicted in Fig. 1a as the Cl^−^ % increased. Fig. S1a† shows the intensities of the hydrides peaks from 10° to 35°, are visibly decreasing with increased Cl^−^ percentage, indicating the poor crystallinity of samples and decrease in grain size.^36^ This may also due to the size difference of the doped atoms making the crystal structure to be expand or contract.^37,38^ The SO_4_^2−^ doped samples are presenting a similar pattern in Fig. 1b as the Cl^−^ doped samples. The peaks related to MgO observed to be low intense, and a peak shift is observed in MgO(111), MgO(200), MgO(220), MgO(331), MgO(222), MgO(400), MgO(331), MgO(420), MgO(422) and Mg(OH)_2_(101) similar to the Cl^−^ doped samples. From 2*θ* = 10°–35° the peaks indicate the presence of multiple hydrides as magnesium carbonate hydroxide hydrate (Mg_2_CO_3_(OH)_2_·3H_2_O) (ICDD 00-006-0484), magnesium oxide sulphate hydrate (Mg_6_O_5_SO_4_·8H_2_O) (ICDD 00-008-0280), magnesium malonate hydrate (C_3_H_2_MgO_4_·2H_2_O) (ICDD 00-026-1851) as shown in Fig. S1b.† A peak shift evident with increasing dopant percentage may be due to formation of hydrides and increased dopant amounts. The PO_4_^3−^ doped samples show poor sample match with the MgO in comparison to the Cl^−^ and SO_4_^2−^ doped samples. However, presence of multiple phases of hydrates such as magnesium phosphate (Mg_3_(PO_4_)_2_) (ICDD 01-075-1491), magnesium phosphate hydrate (Mg_3_(PO_4_)_2_·22H_2_O) (ICDD 00-044-0775), magnesium carbonate hydroxide hydrate (Mg_5_(CO_3_)_4_(OH)_2_(H_2_O)_4_) (ICDD 01-070-0361) and magnesium oxalate (MgC_2_O_4_) (ICDD 00-026-1222), matching with the samples shown in Fig. S1c† can be observed. The PO_4_^3−^ doped samples, indicate the dissolution of both MgO and Mg(OH)_2_ phases and the formation of magnesium oxalate (MgC_2_O_4_) and magnesium phosphate hydrate (Mg_3_(PO_4_)_2_·22H_2_O) in comparison to other two dopants. It is evident from the XRD data that of hydrate formation of hydrates increases from Cl^−^, SO_4_^2−^ to PO_4_^3−^.

However, doped MgO–Mg(OH)_2_ sample peaks with low intensity and wide widths reflect amorphous-like structures with poor crystallinity and defects. Studies^26^ suggest that, generally, structure basic sites favor reversible CO_2_ sorption represented in the following form:Mg–O_(s)_ + CO_2(g)_ → Mg–O–CO_2(ad)_

Perhaps the increase of basic sites with the addition of dopants may result in better adsorption than of without the dopants. Furthermore, due to large number of the hydrides and carbonates present in the samples as explained in the earlier sections from the XRD data, hydrides are expected to anchor large number of hydrogen bonds (H-bonds) from the water molecules on the sample surfaces in all cases (Cl^−^, SO_4_^2−^, and PO_4_^3−^ doped) which eventually form bonds with CO_2_*via* chemisorption.

### Morphological properties

**Fig. 2:**
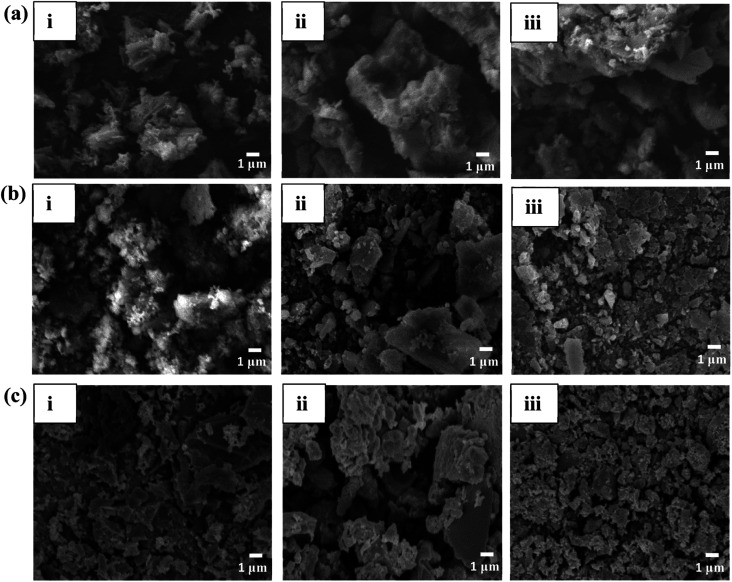
SEM analysis of (a) Cl^−^ doped (b) SO_4_^2−^ doped (c) PO_4_^3−^ doped sample where, (i), (ii) and (iii) are 1%, 5% and 10% dopants in each sample.

The SEM image analysis for the 1%, 5% and 10% Cl^−^ doped MgO samples reveals a combination of rod like and sheet like structures shown in Fig. 2a. This may due to grounding of the involvement of Mg(OH)_2_ utilizing the calcination temperatures of 300 °C and 500 °C XRD data, the hydrate formation to start the competition between H_2_O and CO_2_ for surface cites on the nanocomposite, was validated by the XRD results. Collected powders thoroughly after calcination. The formation of rods indicates a 1-dimensional heterogeneously growth of MgO. The 5% Cl^−^ doped MgO displayed sheets like The adsorption/desorption curves present that even though the large number of hydrates present the 10% Cl^−^ doped sample was stable at 30 °C. Therefore, the hydrates may aided the better performance of the Cl^−^ doped nano composites.^29^

Structures with uniform surfaces, with a typical 2-dimensional diffusion mode. The 10% Cl^−^ doped MgO showed a similar structure to 5% Cl^−^ doped MgO presenting sheet-like uniform structure. The morphology of the SO_4_^2−^ doped MgO samples shown in Fig. 2b, displayed sheet-like structures. However, increasing SO_4_^2−^ concentrations, the grain size was observed to be decreasing. The variations in morphology are comparable to those of MgO developed from Mg(OH)_2_ using various alkali salts when dopant concentrations increase from 1, 5, and 10 wt% Cl^−^.^6^

The loss of water during the breakdown of Mg(OH)_2_ creates a porous structure that will filled with the newly generated MgO particles.^6^ The PO_4_^3−^ doped MgO samples in Fig. 2c also observed to be sheet-like structures under the similar conditions, indicating a strong presence of heterogeneously grown hydrates which support by the XRD data. The morphology of the PO_4_^3−^ doped MgO samples are varied from those of the Cl^−^ and SO_4_^2−^ doped MgO samples. The sheet like structures provide a room to trap CO_2_ in between the layers. The tailored surface chemistry by the dopants may form more defects and vacancies on and in between the sheet like structures, aiding to CO_2_ adsorption.^39^

### CO_2_ capture performance

**Fig. 3:**
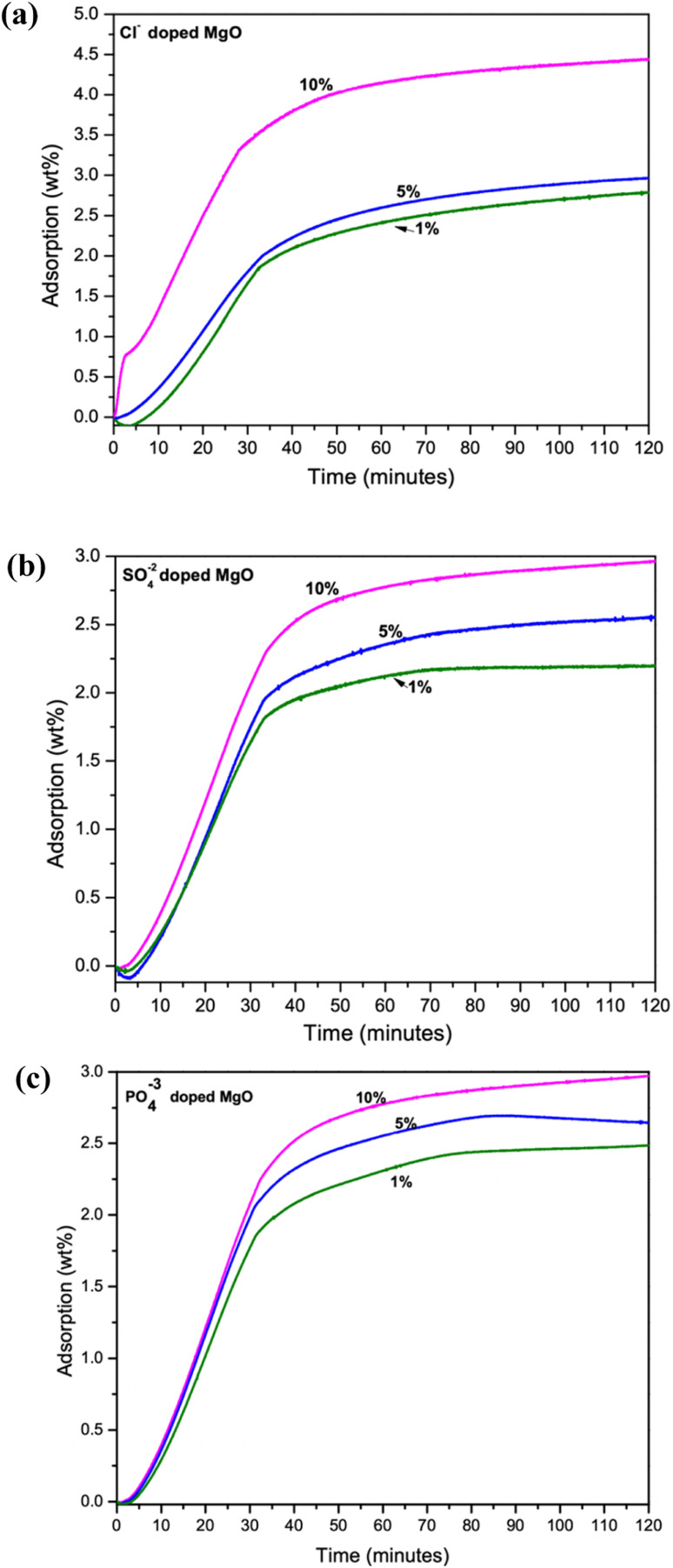
TGA analysis of CO_2_ adsorption capacity of 1%, 5% and 10% doped samples (a) Cl^−^ doped (b) SO_4_^2−^ doped and (c) PO_4_^3−^ doped MgO–Mg(OH)_2_ composites.

The CO_2_ adsorption performance of the samples were measured using a TGA analyzer. The experiment carried out at room temperature (30 °C) To avoid inaccuracies during the analysis, 5–6 mg of samples was used throughout the study. At the start of the measurement, samples were pre-calcined at 150 °C for 60 min while being pumped with high purity N_2_ at a rate of 40 mL min^−1^ and a ramp rate of 10 °C min^−1^. A steady flow of CO_2_ gas (1 atm, 40 mL min^−1^), exposed to record the CO_2_ capture capacity for 1.5 h. Maximum adsorption capacity at 1.5 h adsorption of 4.59 wt% was given by the 10% Cl^−^ doped sample. While 5% Cl^−^ and 1% Cl^−^ doped samples were captured only 2.79 wt% and 2.97 wt% respectively, as shown in Fig. 3a. However, the SO_4_^2−^ and PO_4_^3−^ doped samples recorded low adsorption capacities as shown in Fig. 3b and c, respectively. The SO_4_^2−^ doped samples recorded its maximum adsorption for the 10% doped samples. Adsorption capacity of the samples were observed to be reduced with decreasing dopant percentage.

The PO_4_^3−^ doped samples were also followed the similar trend shown in Fig. 3c. However, the disappearance of the MgO–Mg(OH)_2_ phase in PO_4_^3−^ doped samples may account for their much lower CO_2_ adsorption among the three dopants. Therefore, it is evident that increasing dopant percentage increases CO_2_ adsorption capacities of the synthesized composites. However, the CO_2_ adsorption performance of SO_4_^2−^ and PO_4_^3−^ doped samples are deficient in comparison to the Cl^−^ doped samples.

### Properties of Cl^−^ doped MgO–Mg(OH)_2_ composites

From the CO_2_ adsorption capacity, it is evident that Cl-doped samples performed better in comparison to SO_4_^2−^ and PO_4_^3−^ doped composites. Therefore, further chemical equilibria calculations for the possible bulk reactions for the Cl^−^-doped systems were carried out by using FactSage^40^ commercial software.

(1) 1 wt% MgCl_2_1.698 (mol) Mg(OH)_2_ + 0.011 (mol) MgCl_2_ + 0.112 (mol) H_2_O + 0.112 (mol) CO_2_ = 1.688 (mol) MgO + 0.018 (mol) Mg(OH)Cl + 0.0008MgCO_3_ + gas phase

(2) 5 wt% MgCl_2_1.629 (mol) Mg(OH)_2_ + 0.053 (mol) MgCl_2_ + 0.104 (mol) H_2_O + 0.104 (mol) CO_2_ = 1.579 (mol) MgO + 0.102 (mol) Mg(OH)Cl + 0.0001MgCO_3_ + gas phase

(3) 10 wt% MgCl_2_1.543 (mol) Mg(OH)_2_ + 0.105 (mol) MgCl_2_ + 1 (mol) H_2_O + 1 (mol) CO_2_ = 1.44 (mol) MgO + 0.21 (mol) Mg(OH)Cl + gas phase

The CO_2_ threshold (at an equal CO_2_ to H_2_O ratio) for the formation of MgCO_3_ is respectively >1.0, 0.104, and 0.112 (moles) for a sample of 100 g. Therefore, it is unlikely that MgCO_3_ is stable in samples with 10 wt% MgCl_2_ and 90% Mg(OH)_2_ but the 5 wt% and 1 wt% MgCl_2_ samples, because of the much lower (≪0.5 atm) CO_2_ level in air. It is interesting that based on thermodynamic calculation, the 5 wt% MgCl_2_ sample has the lowest CO_2_ threshold, which correlated well with the heights of the glass phase XRD peaks from 10° to 35°. To further invest if hydrates form in the 5 wt% MgCl_2_ sample at 25 °C, the following calculation is obtained:

(4) 5 wt% MgCl_2_1.629 (mol) Mg(OH)_2_ + 0.053 (mol) MgCl_2_ + 0.104 (mol) H_2_O + 0.000001 (mol) CO_2_ = 1.603 (mol) Mg(OH)_2_ + 0.053 (mol) Mg(OH)Cl + 0.0001 MgCO_3_ + 0.026 MgCl_2_(H_2_O)_4_ + gas phase

At lowered CO_2_ mole environment (which replicate the CO_2_ levels in air) and at 25 °C, a new hydrate phase, MgCl_2_(H_2_O)_4_, is formed. This new formed phase supports the assumption that hydrate glass phases may be represented by the XRD peaks from 10° to 35° (Fig. S1a†). Therefore, from thermodynamics, it is evident that hydration is a competition process to CO_2_ adsorption. The surface area is one of the significant properties of adsorbents as it influences the amount of gas capture capacities.

**Fig. 4:**
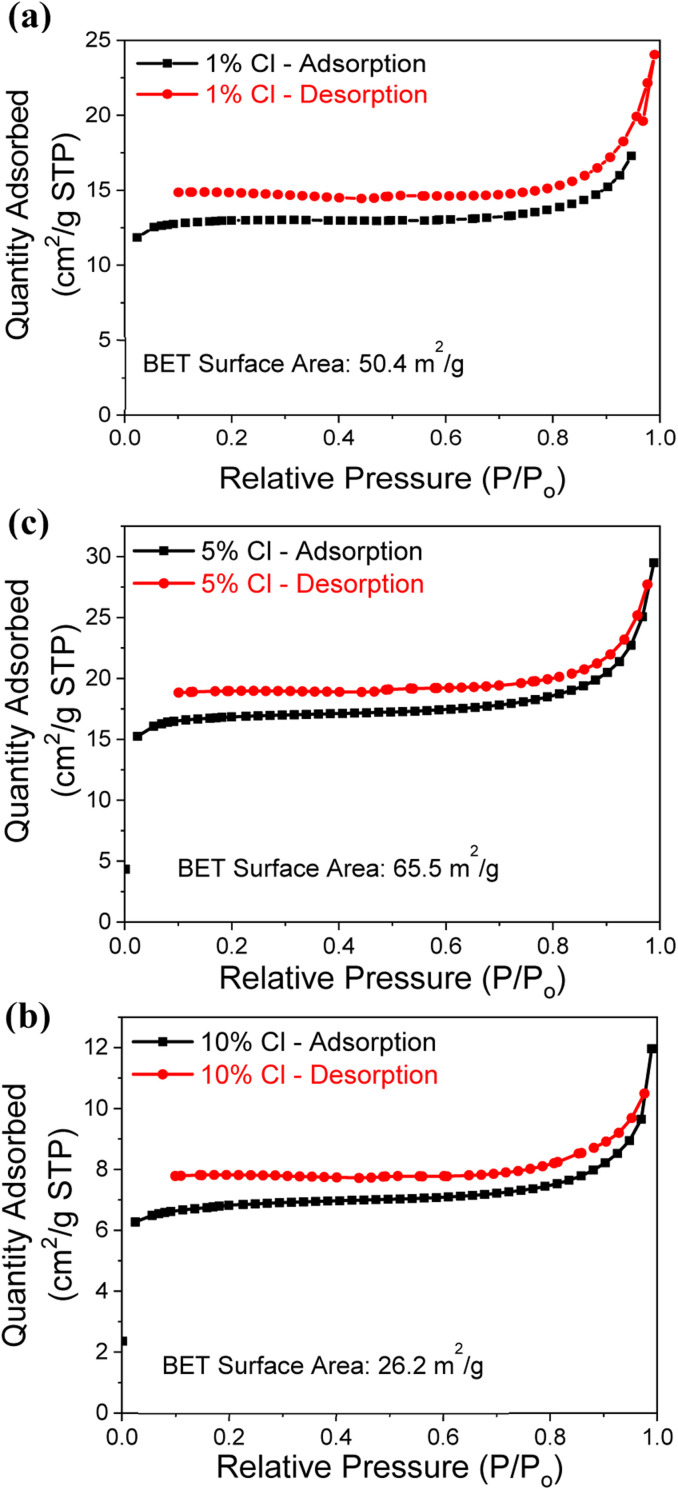
N_2_ adsorption–desorption isotherms analysis for the (a) 1%, (b) 5% and (c) 10% Cl^−^ doped samples.

Since the Cl^−^ doped samples recorded the higher CO_2_ capture capacity, the N_2_ adsorption–desorption isotherms are measured to determine the surface area of samples, as illustrated by Fig. 4a–c and summarized in Table 1. Low pressure and 200 °C were used during the analysis, with 0.3 g of powder from each Cl^−^ doped samples. All 3 samples (1%, 5% and 10% Cl^−^ doped) exhibited a type II isotherm with a hysteresis loop of type H3 according to the IUPAC classification.^41^ The H3 hysteresis typically caused by samples that are agglomerated and have the sheet like structures with flexible pores.^25,42^ The N_2_ adsorption observed to be decreased with increasing dopant percentage. The 5% Cl^−^ doped MgO sample determined to have the higher specific surface area of 65.5 m^2^ g^−1^ compared to the other samples. This observation may be due to the presence of hydrate phases (2 theta angles from 10°–35° in Fig. S1b†) reaches the maximum at 5% Cl^−^. Although the 10% Cl^−^ doped MgO sample presented the higher CO_2_ capture capacity it shows the lowest specific surface area of 26.2 m^2^ g^−1^.

**Table 1:** BET surface area of Cl^−^ doped MgO samples

Sample	BET surface area (m^2^ g^−1^)
1% Cl^−^	50.4
5% Cl^−^	65.5
10% Cl^−^	26.2

**Fig. 5:**
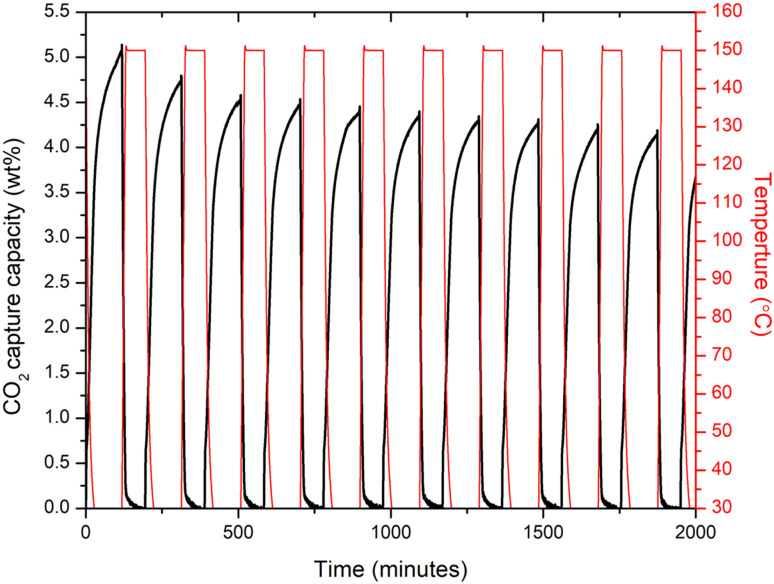
CO_2_ adsorption/desorption curves of 10% Cl doped sample over 10 cycles at 30 °C (adsorption condition: 30 °C, 1 atm, 100% pure CO_2_, 1.5 h. Desorption condition: 30 °C, 1 atm, 100% pure N_2_, 1 h).

Recovery and long term stability of an adsorbent material after CO_2_ capture is another important characteristics that needs to be further analyse for them to apply in real conditions. Therefore, the 10% Cl^−^ doped sample which performed better in comparison to other doped samples, subjected to adsorption/desorption cycles as shown in Fig. 5. The 10% Cl^−^ doped sample's CO_2_ absorption at 30 °C was discovered to fall from 5.12 to 4.19 wt% during 10 cycles, demonstrating the novel adsorbent's strong long-term adsorption/desorption stability. In comparison, to the recent studies,^28,43^ the 10% Cl^−^ doped sample, the drop rate of CO_2_ adsorption capacity over 10 adsorption/desorption cycles at 30 °C was about 18%, suggesting a better cycle stability indicating the adsorbent material may perform well in capturing the atmospheric CO_2_ long term.

As the 5% Cl^−^ doped sample observed to have the higher surface area, the same sample was then subjected to analyze the gas sensing capability at high temperature conditions. Alpha-terpineol was used (Sigma-Aldrich) as the binder and 0.01 g of 5% Cl^−^ doped powder was mixed with 0.01 ml of alpha-terpineol. Then, the mixed solution was coated on the electrode substrate using the screen-printing method. Subsequently, the samples were dried at 60 °C for 30 min. Finally, to remove remaining solvents, the samples were heat-treated at 250 °C for 1 h in air.

The properties of the 5% Cl^−^ doped sample sensor, interdigitated titanium and platinum electrodes were sequentially deposited by direct current (DC) sputtering on the substrate with thicknesses of 50 nm and 200 nm, respectively. For the gas sensing measurements, the constructed sensor was electrically coupled to a Keithley 2400 source meter. Data from dynamic sensing were captured with a constant DC bias of 1 V. The 5% Cl^−^ doped sample gas sensor's CO_2_ detection capabilities were tested under gas concentrations of 50–5000 ppm at temperatures ranging from 25–300 °C. The chemisorption of CO_2_ renders the 5% Cl^−^ doped composite a subpar CO_2_ sensing material. Table 2 summarizes the CO_2_ gas response in 5% Cl^−^ doped composites. The measurement at 300 °C detects the CO_2_ at 500 ppm levels because of the thermal decomposition temperature to MgCO_3_ is about 327 °C. However, at low temperature the sensors are not performing well.

**Table 2:** CO_2_ gas responses at different sensing temperatures for the 5% Cl^−^ doped sample

Temperature (°C)	5000 ppm	1500 ppm	500 ppm	150 ppm	50 ppm
200	1.017	1	1	1	1
250	1.022	1.014	1	1	1
300	1.013	1.009	1.006	1	1

### Theoretical analysis of CO_2_ adsorption on MgO and Mg(OH)_2_

**Fig. 6:**
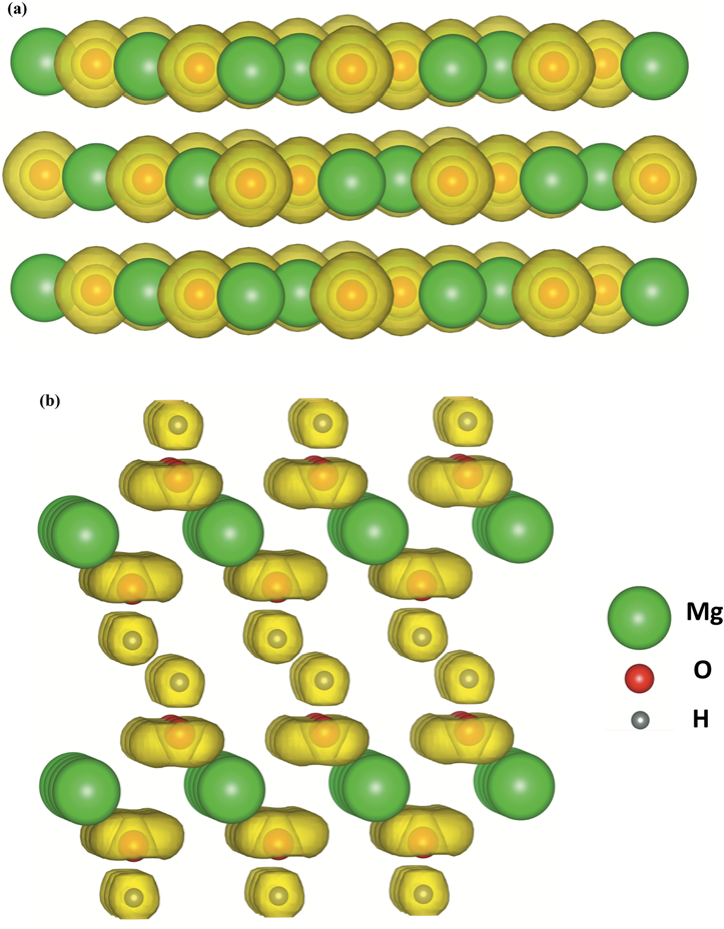
Valence ELF iso-surfaces evaluated at ELF = 0.80 for (a) MgO and (b) Mg(OH)_2_.

Adsorption of CO_2_ molecules on the most stable MgO and Mg(OH)_2_ surfaces are simulated using first-principles calculation respectively to determine the surface affinity to CO_2_ molecules. The electronic structure of the adsorbent is crucial to its affinity. Fig. 6 visualizes the electronic density of MgO and Mg(OH)_2_ in terms of valence electron localization function (ELF) iso-surface at ELF = 0.80. Distinctive discrepancy in bonding characteristic is observed between MgO and Mg(OH)_2_. The localized electron is spherically distributed in the O^2−^ anion in MgO, indicating a pure ionic bond. Since CO_2_ is a Lewis acid molecule and tends to accept electrons, the accumulation of electrons on the O^2−^ anions are beneficial to the attraction of CO_2_ molecules to MgO surface containing O atoms. As to the OH^−^ anion in Mg(OH)_2_, the strong covalent contribution to O–H bonding caused by the deep overlapping of the valence orbitals of oxygen and hydrogen is established in the vertical direction. In contrast, the regular torus-shaped iso-surface in the planar directions is formed from contributions of localized lone-paired electrons. Due to the oriented vertical deformation of the localized electronic cloud from sphericity, this covalency has partially polar character with negative O ion and position H ion. The positively charged H cations are obstructive to the attraction of CO_2_ molecules to Mg(OH)_2_ surface comprised by H atoms. The distinctive discrepancy of affinity to CO_2_ molecules in MgO and Mg(OH)_2_ surfaces gives rise to significantly different CO_2_ adsorption behaviors.

**Fig. 7:**
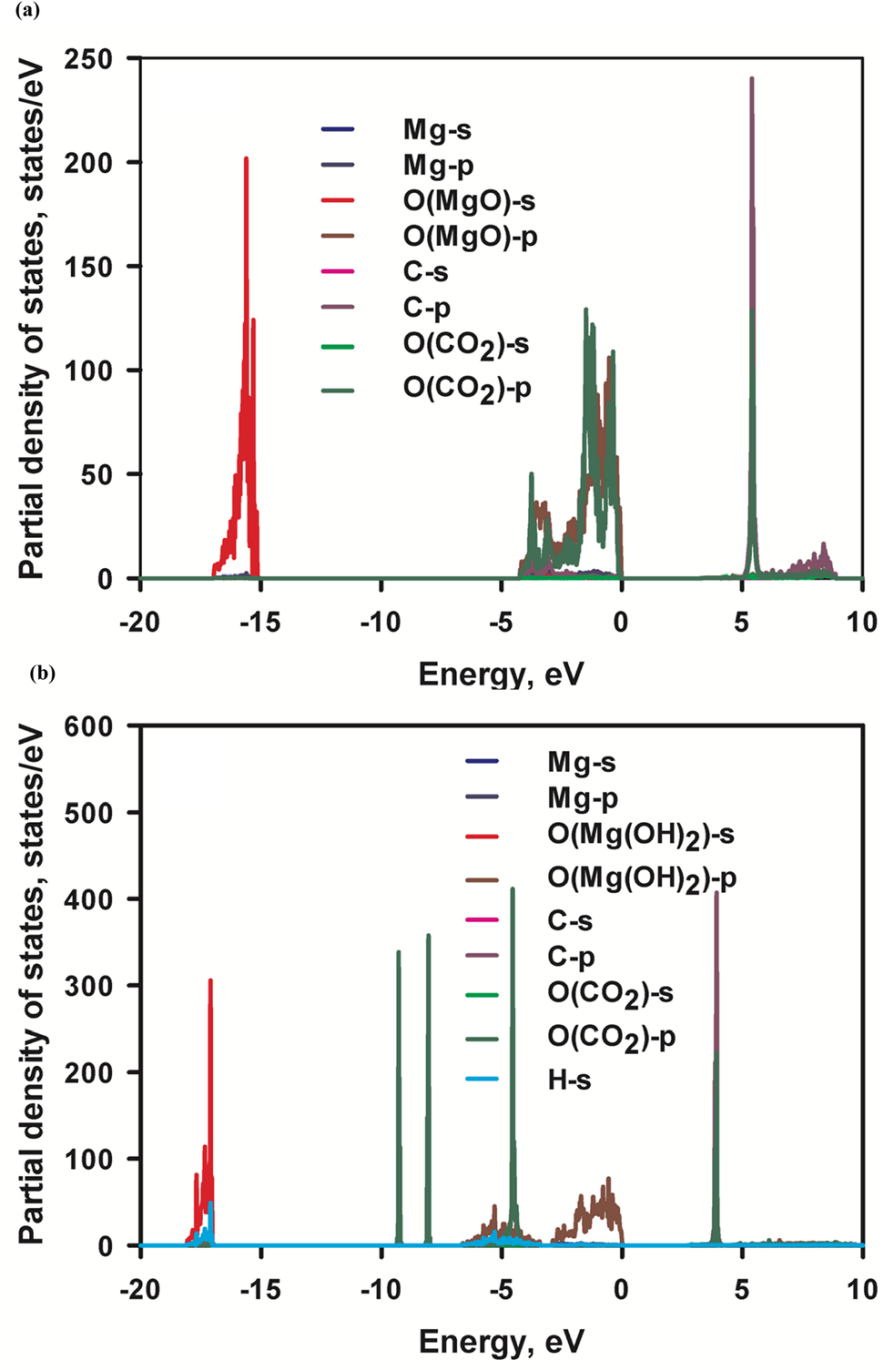
Partial density of states (PDOS) of (a) MgO surface with adsorbed CO_2_ and (b) Mg(OH)_2_ surface with adsorbed CO_2_, respectively. The PDOS of C and O of CO_2_ in MgO is magnified by 50 and 25 respectively, while the PDOS of C and O of CO_2_ in Mg(OH)_2_ is magnified by 25 and 12.5 respectively.

Adsorption of CO_2_ molecules on the most stable (001) MgO and Mg(OH)_2_ surfaces are simulated respectively with their corresponding partial density of states (PDOS) are presented in Fig. 7. Appreciable hybridization of p orbitals between C atom and O (MgO) atoms and trivial hybridization of p orbitals of O (CO_2_) atom and s orbital of Mg atoms are observed in MgO surface, indicating strong interaction between C and O (MgO) atoms. This is consistent with the inferred strong affinity of MgO to CO_2_ molecules. The interaction leads to the strong physical adsorption of CO_2_ to MgO surface. This is evidenced by the calculated CO_2_ adsorption energy −0.48 eV. No perceptible hybridization between C atom and O(Mg(OH)_2_) atom or between O(CO_2_) atom and Mg atom in Mg(OH)_2_ surface is noted in Fig. 6b, being consistent with the weak affinity of Mg(OH)_2_ to CO_2_ molecules. Therefore, the adsorption of CO_2_ molecules on Mg(OH)_2_ surface is entirely by weak dispersion forces. This agrees with the calculated marginal CO_2_ adsorption energy −0.046 eV. The energy barrier from physisorption to chemisorption is generally defined as −0.52 eV. Therefore, MgO and Mg(OH)_2_ can be classified as the strong end and the weak end of the physical adsorption of CO_2_ respectively. Consequently, it can be assumed that MgO is CO_2_-philic adsorbent while Mg(OH)_2_ is CO_2_-phobic adsorbent.

One intriguing feature of Mg(OH)_2_ is that it is a chemically versatile solid hydroxide. It can be dehydrated into simple oxide and water under heat,^44^
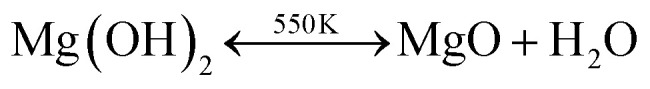
vice versa, it can be synthesized *via* the backward reaction with the thermal mechanism represented as the following:^45^H_2_O_dissolved_ + O_structure_^2−^ → (OH^−^)_structure_ + (OH^−^)_structure_

The transformation from MgO to Mg(OH)_2_ is mainly achieved by structural rearrangements of O^2−^ ions. The large electronegativity differences between Mg and O, the linking of Mg^2+^ and O^2−^ ions *via* charge-transfer gives rise to strong Mg–O ionic bonds, then the direct binding of water molecules to metal centres becomes impossible. Therefore, it renders us an opportunity to synthesize interweaved MgO–Mg(OH)_2_ composites to mimic Namib Desert Beetles, whose surface texture is comprised of wax free hydrophilic bumps and waxy hydrophobic valleys, for efficient water collection and droplet formation.^46^ With controlling of calcination temperature, it is potential to synthesize surface texture with interweaved CO_2_-philic MgO part for efficient CO_2_ capture by physical adsorption and CO_2_-phobic Mg(OH)_2_ part for massive CO_2_ storage by chemical reaction. Our first-principles calculations on Mg(OH)_2_ monolayer further suggest that doping and charge be additional approaches to adjust the CO_2_-phobicity of Mg(OH)_2_.^47^

## Conclusion

In conclusion, our study has successfully synthesized, characterized, and validated a range of anion-doped MgO–Mg(OH)_2_ nanocomposites for ambient CO_2_ adsorption. By optimizing the composites, we were able to tailor the CO_2_-phobicity in Mg(OH)_2_, effectively inhibiting carbonate production and enhancing CO_2_ diffusion through the nanocomposites. Among the doped samples, the Cl^−^ doped composite exhibited efficient CO_2_ adsorption at 30 °C and demonstrated stability throughout multiple adsorption/desorption cycles. On the other hand, the CO_2_ adsorption studies for the PO_4_^3−^ doped samples indicated a lack of facilitation for CO_2_ adsorption at room temperature, while the SO_4_^2−^ samples showed relatively favorable results. Furthermore, the introduction of dopants plays a vital role in understanding the impact of MgO-based sorbents on improving CO_2_ capture properties. This work expands the design space, offering new opportunities for the development of enhanced and cost-effective CO_2_ adsorbents based on magnesium minerals. This expansion includes transitioning from the binary MgO–H_2_O system to ternary MgO–H_2_O–MgX or even quaternary MgO–H_2_O–MgX1–MgX2 systems. By exploring these avenues, we can further enhance the performance and efficiency of magnesium-based adsorbents for CO_2_ capture.

## Conflicts of interest

The authors declare no conflict of interest.

## Author contributions

Conceptualization, P. W. and H. L. S.; methodology H. L. S.; validation, H. L. S., C. L. and S. W.; investigation, H. L. S.; data curation, H. L. S., S. W., J. Y. K.; writing – original draft preparation, P. W., S. W. and H. L. S.; writing – review and editing, H. L. S., P. W., S. W., S. S. K., and K. B.; all authors have read and agreed to the published version of the manuscript.

## Supplementary Material

RA-013-D3RA04080A-s001
