# Supplementary material for: Enhancing MgO efficiency in CO2 capture: engineered MgO/Mg(OH)2 composites with Cl−, SO42−, and PO43− additives
Source: RSC Adv. 2023 Sep 20;13(40):27946–55. doi: 10.1039/d3ra04080a (PMC10509748; doi:10.1039/d3ra04080a)
Supplement: RA-013-D3RA04080A-s001 [file RA-013-D3RA04080A-s001.pdf]

Electronic Supplementary Material (ESI) for RSC Advances.
This journal is © The Royal Society of Chemistry 2023

Enhancing MgO Efficiency in CO_2 Capture: Engineered MgO/$Mg(OH)_2$ Composites with Cl^-, SO_4^{2-}, and PO_4^{3-} Additives

Hasanthi L. Senevirathna[a], Shunnian Wu[a], Cathie Lee[a], Jin-Young Kim[b], Sang Sub Kim[b], Kewu Bai[c], Ping Wu*[a]

[a]Entropic Interface Group, Engineering Product Development, Singapore University of Technology and Design, 8 Somapah Road, Singapore 487372.
[b]Department of Materials Science and Engineering, Inha University, Incheon 22212, Korea.
[c]Institute of High Performance Computing, Agency for Science, Technology and Research, Fusionopolis Way, #16-16 Connexis, Singapore 138632, Singapore.

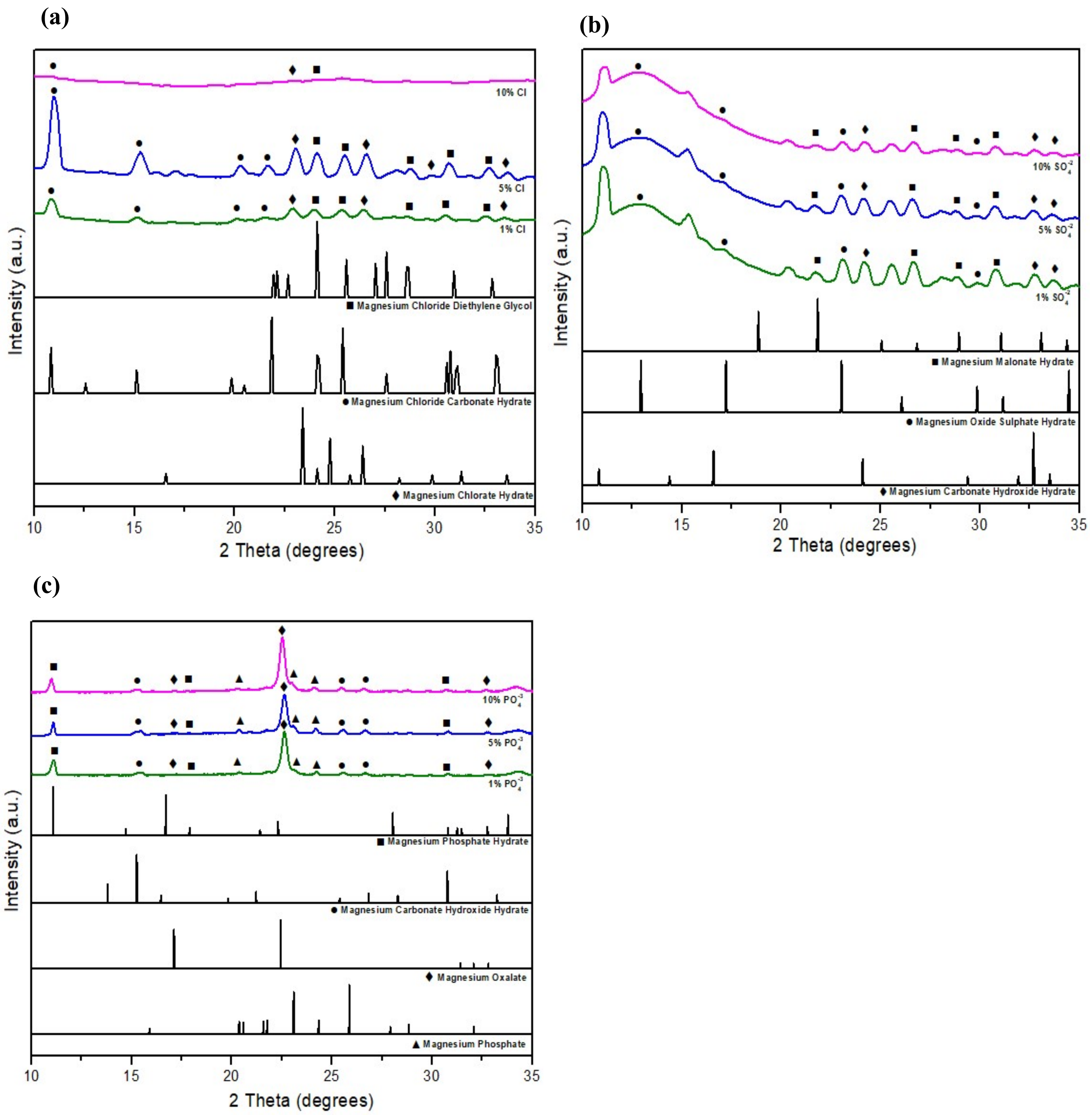

Fig. S1 (a) Magnification of the broader peaks 2θ = 10˚- 35˚ associated with hydrates in the samples with increasing Cl^- percentage. (b) Magnification of peaks 2θ = 10˚ - 35˚ associated with hydrates related to SO_4^{2-} in the samples with increasing SO_4^{2-} percentage. (c) Magnification of peaks 2θ = 10˚ - 35˚ associated with hydrates related to PO_4^{3-} in the samples with increasing PO_4^{3-} percentage.
